# Reappraising Social Insect Behavior through Aversive Responsiveness and Learning

**DOI:** 10.1371/journal.pone.0004197

**Published:** 2009-01-14

**Authors:** Edith Roussel, Julie Carcaud, Jean-Christophe Sandoz, Martin Giurfa

**Affiliations:** Research Center on Animal Cognition (UMR5169), Centre National de la Recherche Scientifique (CNRS) - University Paul-Sabatier, Toulouse, France; CNRS, France

## Abstract

**Background:**

The success of social insects can be in part attributed to their division of labor, which has been explained by a response threshold model. This model posits that individuals differ in their response thresholds to task-associated stimuli, so that individuals with *lower* thresholds specialize in this task. This model is at odds with findings on honeybee behavior as nectar and pollen foragers exhibit different responsiveness to sucrose, with nectar foragers having *higher* response thresholds to sucrose concentration. Moreover, it has been suggested that sucrose responsiveness correlates with responsiveness to most if not all other stimuli. If this is the case, explaining task specialization and the origins of division of labor on the basis of differences in response thresholds is difficult.

**Methodology:**

To compare responsiveness to stimuli presenting clear-cut differences in hedonic value and behavioral contexts, we measured appetitive and aversive responsiveness in the same bees in the laboratory. We quantified proboscis extension responses to increasing sucrose concentrations and sting extension responses to electric shocks of increasing voltage. We analyzed the relationship between aversive responsiveness and aversive olfactory conditioning of the sting extension reflex, and determined how this relationship relates to division of labor.

**Principal Findings:**

Sucrose and shock responsiveness measured in the same bees did not correlate, thus suggesting that they correspond to independent behavioral syndromes, a foraging and a defensive one. Bees which were more responsive to shock learned and memorized better aversive associations. Finally, guards were less responsive than nectar foragers to electric shocks, exhibiting higher tolerance to low voltage shocks. Consequently, foragers, which are more sensitive, were the ones learning and memorizing better in aversive conditioning.

**Conclusions:**

Our results constitute the first integrative study on how aversive responsiveness affects learning, memory and social organization in honeybees. We suggest that parallel behavioral modules (e.g. appetitive, aversive) coexist within each individual bee and determine its tendency to adopt a given task. This conclusion, which is at odds with a simple threshold model, should open new opportunities for exploring the division of labor in social insects.

## Introduction

The origin of social life represents a major evolutionary transition which has occurred repeatedly across many lineages [1]. Social insects, with their complex colony organization, division of labor and sophisticated communication systems, provide an ideal model for studying the biological bases of social organization [2] Among social insects, the honeybee (*Apis mellifera*) constitutes a well-studied case of social organization which has attracted during decades the interests of researchers [3]. Honeybees are highly eusocial as they exhibit reproductive division of labor (with sterile and reproductive castes), generational overlap and cooperative brood care [2].

The ecological and evolutionary success of bees and other social insects can in part be explained by their division of labor, in which individuals specialize in performing different tasks [4], and by their learning and memory capabilities which provide a basis for responding in an adaptive way to a changing environment. Different models have been proposed to explain the origin of division of labor [5]. Among these, the response threshold model is widely accepted and postulates that individuals differ in their response threshold to task-associated stimuli [6], [7]. This model has received strong empirical support in many taxa and contexts. For instance, nurse bees are more sensitive, and thus more responsive, to the stimulation provided by larvae than guards so that they specialize as brood tenders [8]. In other words, individuals highly sensitive to a given stimulus are good candidates for becoming specialized in tasks involving such a stimulus [9]. Sensitivity can be evaluated by measuring response thresholds in well-defined experimental protocols. In honeybees, the existence of specialization in nectar or pollen collection has led to a series of studies which constitute the best studied case of how variations in behavioral responsiveness may result in task specialization [10]. Indeed, differences between nectar and pollen foragers have been accounted for by the occurrence of different response thresholds to sucrose stimulation. Such thresholds are measured by the proboscis extension reflex (PER), the innate response of a hungry bee to stimulation of its antennae with a drop of sugar solution of increasing concentration [11]. The lowest concentration which the bee can distinguish from water defines its sucrose responsiveness threshold. Interestingly, nectar foragers exhibit higher thresholds (i.e. lower responsiveness) than pollen foragers, which exhibit lower thresholds and thus higher responsiveness. Although this difference may appear counterintuitive at a first sight, its adaptive value for bees could be that nectar foragers are more selective when collecting nectar, and will therefore provide the highest energy gain to the colony. Sucrose responsiveness thresholds vary with a series of factors such as age, caste, sex, [12], foraging experience, genotype, feeding [13], season [14], stress (handling), hormone levels, pheromones [15], among others.

Sucrose responsiveness thresholds have a further behavioral consequence, which is of fundamental importance for individual success: they affect learning and memory performance. As mentioned above, honeybees and other social insects have extremely well-developed learning and memory capabilities [16]. In controlled protocols in which harnessed bees learn to associate an olfactory or tactile stimulus with sugar reward, bees that are more responsive to sucrose learn faster and show higher performance than less responsive bees [14], [17]–[22], and consequently remember better the learned appetitive associations [21], [22].

The plethora of studies on sucrose responsiveness has led to the general idea that this unique behavioral trait can explain diverse behavioral responses [23] to stimuli as different from sugar as odors or light [24], [21]. Indeed, Page et al. [10] state that “*bees who are sensitive to sucrose are also sensitive to stimuli of other modalities*” so that “*sucrose responsiveness can be used as a robust indicator for general differences of processing information in the central nervous system*”. This conclusion has dramatic consequences for current theories on the evolution of sociality as it defines a new theoretical framework for interpreting the division of labor. In particular, the suggestion that bees sensitive to stimuli of a given sensory modality exhibit, at the same time, high sensitivity to all other stimuli, even belonging to different sensory modalities, is puzzling because it may imply a suboptimal division of labor, which in theory critically depends on the existence of different sensitivities for different stimuli.

This assumption could be, however, erroneous as the behavioral traits that have been related so far to sucrose responsiveness all have in common, an appetitive framework, i.e. are related to foraging behavior. In a drastically different framework, in which stimuli possess a hedonic value different from sucrose or its related context, would the theory mentioned above still be valid? In other words, do bees that exhibit high responsiveness to sucrose also display high responsiveness to an aversive stimulus? To answer this question, we determined whether or not sucrose responsiveness correlates with responsiveness to electric shocks of varying voltage. Harnessed bees extend reflexively their sting (sting extension reflex or SER) when stimulated with a mild electric shock [25]–[29]. Like PER for sucrose, SER allows, therefore, direct quantification of response thresholds to a stimulus that, in this case, is fully independent of a foraging context.

As mentioned above, sucrose responsiveness directly affects appetitive learning and memory performances. In a similar way, does responsiveness to an aversive stimulus have the same effect on aversive learning performances? The advent of a new protocol for olfactory aversive conditioning of SER [30] may provide answers to this question. In this protocol, harnessed bees learn to associate an odorant with a mild electric shock (7.5 V) so that they respond to the punished odorant with a SER. It therefore allows asking whether or not bees that are more sensitive to electric shocks also learn and remember better the aversive olfactory associations. Moreover, it is also important to determine whether shock responsiveness also differs between different task-specialized bees. Like nectar vs. pollen foragers, which differ in their sucrose responsiveness and thus in their appetitive learning capabilities, do guard and foragers bees differ in their shock responsiveness and therefore in their aversive learning capabilities?

We show here that sucrose responsiveness is not correlated with shock responsiveness, thus providing the first demonstration that sucrose responsiveness does not account for sensitivity to all sensory modalities, especially when stimuli differ in their hedonic value. Shock responsiveness is however correlated with aversive learning and retention performances, thus proving that irrespective of the hedonic nature of the reinforcement, bees learn and remember better when they are particularly sensitive to the reinforcement used. Finally, we show that nectar foragers are more sensitive to shocks than guards and perform better in aversive conditioning.

We propose that sensitivity to aversive stimuli may control defensive behavior as sensitivity to sucrose controls foraging behavior. These results suggest the existence of parallel modules determining honeybees' behavior and open new opportunities for exploring the division of labor in social insects.

## Results

### Experiment 1: Do sucrose and shock responsiveness correlate?

**Figure 1:**
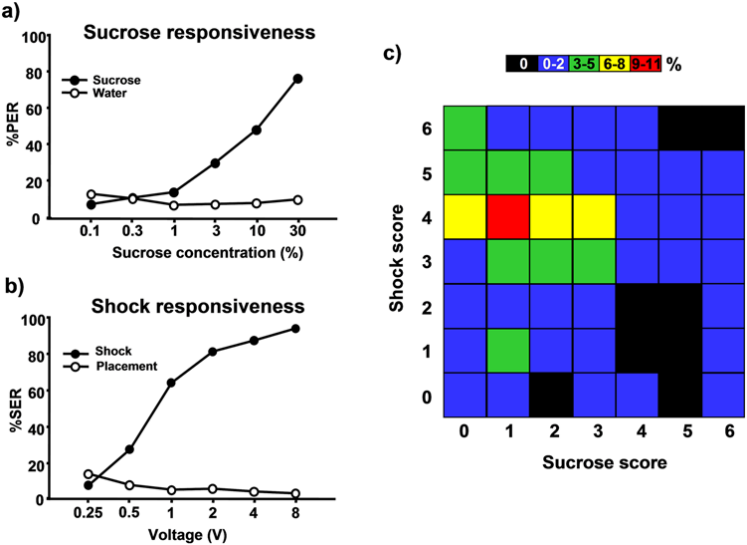
Relationship between sucrose and shock responsiveness in honeybees. a) Sucrose responsiveness. Black circles, % of PER to a series of sucrose solutions of increasing concentration (n = 198); white circles, % of PER of the same bees to the presentation of water (control). Bees increased their response to sucrose solution of increasing concentrations. b) Shock responsiveness of the same bees. Black circles, % of SER to a series of shocks of increasing voltage; white circles, % of SER of the same bees to placements in the same setup without shock delivery (control). Bees increased their response to shocks of increasing voltage. c A 7×7 matrix of correlation between sucrose and shock responsiveness scores in the same bees. Scores varied from 0 (no response to any stimulus tested in the series) to 6 (responses to all six stimuli of the series). Colors assigned to each box represent the percentage of bees exhibiting a particular combination of sucrose and shock responsiveness scores. No significant correlation exists between sucrose and shock responsiveness scores (R = −0.03; t (N-2) = −0.42; NS).

This experiment was designed to test the possible correlation between sucrose and shock responsiveness in the same bees. If, as suggested [10], bees that are sensitive to one stimulus are also highly sensitive to other kinds of stimuli, responsiveness to a series of sucrose solutions of increasing concentration should be highly correlated with responsiveness to a series of electric shocks of increasing voltage. To test this hypothesis, we measured in harnessed bees sucrose responsiveness (PER) to a logarithmic series of sucrose solutions of increasing concentration in a first phase, and shock responsiveness (SER) to a series of shocks of increasing voltage in a second phase (n = 94). The reversed sequence (first shock, then sucrose) was employed in another group of bees tested in parallel (n = 104). Neither the responses to the electric shocks (ANOVA for repeated measurements; F_1,196_ = 0.83, NS) nor the responses to the sucrose solutions differed significantly between these two groups (F_1,196_ = 0.05, NS). Furthermore, the interaction between group and stimulus type was also non-significant (F_1,196_ = 0.87, NS for sucrose and F_1,196_ = 0.24, NS for electric shocks), thus confirming that responsiveness to sucrose and shock were unaffected by the order of stimulation. Results were therefore pooled and presented in Fig. 1a and 1b which also shows responses to control stimulations interspersed between sucrose or shock trials. Water stimulation was used as the control for sucrose stimulation, and placements (i.e. positioning of the bees in the stimulator without stimulus delivery) as the control for electric shock stimulation.

As expected, bees significantly increased their appetitive response (PER) to sucrose solutions of increasing concentration (Fig. 1a: F_5,985_ = 161.46, p<0.0001). Similarly, bees significantly increased their aversive response (SER) to electric shocks of increasing voltage (Fig. 1b: F_5,985_ = 278.7, p<0.0001). By contrast, bees decreased their responses both to water (F_5,985_ = 2.65, p<0.05) and to placement (F_5,985_ = 7.63, p<0.0001) along trials, thus excluding the possibility that responses may have been influenced by sensitization induced by sucrose or shock.

Are the bees responding maximally to the highest voltages the ones responding also maximally to the highest sucrose concentrations? To answer this question, we assigned to each bee both a sucrose score and a shock responsiveness score. Scores were quantified as the sum of all responses made along the whole scale of tested stimuli. For example, a bee extending its sting from 0.5 to 8 V, i.e. to five out of the six voltages assayed, had a shock responsiveness score of 5 as it responded to five consecutive voltages. This bee had also a sucrose responsiveness score derived from its response to the six concentrations of sucrose solution. Scores may therefore vary from 0 (no response to any stimulus tested in the series) to 6 (responses to all six stimuli of the series). Results of this analysis can thus be represented as a 7×7 matrix in which one axis is defined by sucrose responsiveness scores and the other axis by shock responsiveness scores (Fig. 1c). Colors assigned to each box represent the percentages of bees exhibiting a particular combination of sucrose and shock responsiveness scores. Figure 1c shows no predictive pattern of responsiveness to the appetitive and aversive stimulations. Indeed, a Spearman rank correlation analysis confirmed the lack of correlation between shock and sucrose responsiveness in honeybees (R = −0.03; t (N-2) = −0.42; NS).

### Experiment 2: Does shock responsiveness determine aversive learning and retention performances?

It has been suggested that bees that are highly sensitive to sucrose show better appetitive learning performances [14], [17]–[22]. Does shock responsiveness affect in a similar way olfactory aversive learning in bees? To answer this question, we determined shock responsiveness scores as above and then divided our bees in two groups according to their scores, a low-responsiveness group (scores 1 to 3) and a high-responsiveness group (scores 4 to 6). On the next day, bees were trained in a differential conditioning procedure (6 trials with one odorant paired with shock, or CS+, and 6 trials with an odorant not paired with shock, or CS−). Retention tests with both odors were performed 1 h after the last conditioning trial.

**Figure 2:**
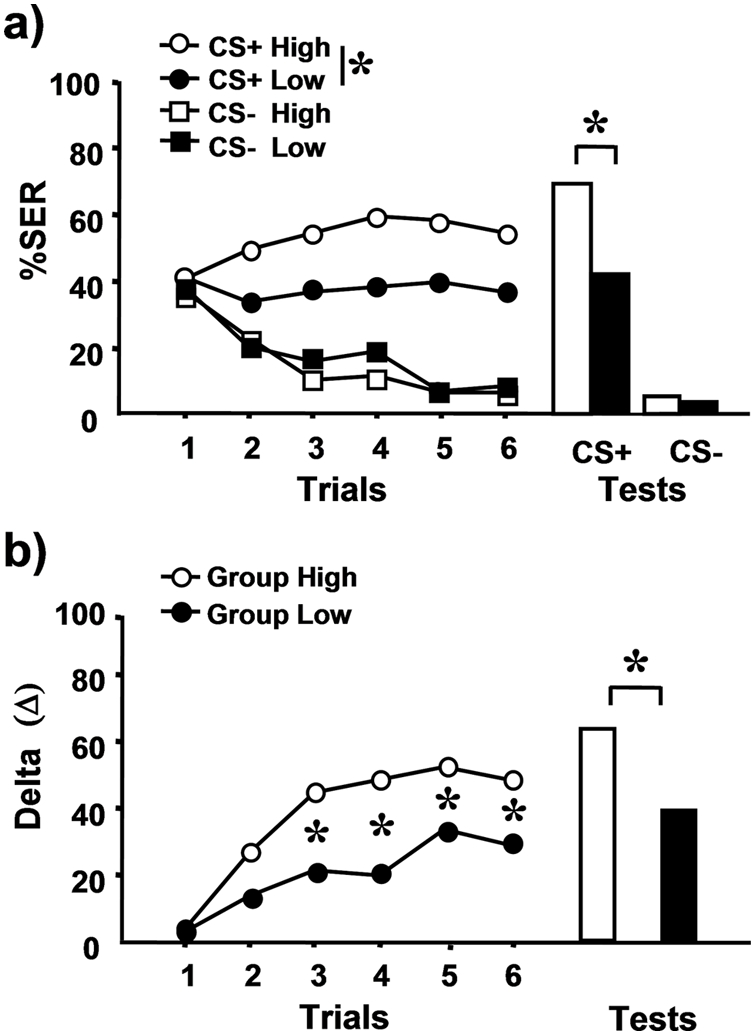
Learning and retention performances in olfactory conditioning of SER as depending on shock responsiveness. a) Black symbols: % of SER in differential conditioning of a low-responsiveness group (scores 1–3; n = 80); white symbols: % of SER in differential conditioning of a high-responsiveness group (scores 4–6; n = 67). Circles: responses to the CS+; squares: responses to the CS−. Both groups learned the differentiation between punished and non-punished odors but bees of the high-responsiveness group achieved better performances than bees of the low-responsive group and remembered better one hour after conditioning (white vs. black bars). b) Delta value (Δ) resulting from the difference between the response to the CS+ and to the CS−, for high-responsiveness bees (white circles) and low-responsiveness bees (black circles). High-responsiveness bees learned and remembered better (white vs. black bars) the discrimination between CS+ and CS−. *: p<0.05.

As observed in the previous experiment, bees significantly increased their aversive response (SER) to electric shocks of increasing voltage (F_5,1980_ = 487.23, p<0.0001; not shown). Bees with low responsiveness scores (scores 1 to 3; n = 67) responded only to higher voltages (2 to 8 V) while bees with high responsiveness scores (scores 4 to 6; n = 80) responded to a broader range of voltages starting with lower ones (0.25, 0.5 or 1 V). Figure 2a shows that both groups learned to discriminate the odorant reinforced with shock from the non-reinforced odorant in the aversive olfactory conditioning protocol (low-responsiveness group: F_1,132_ = 14.4, p<0.0005; high-responsiveness group: F_1,158_ = 65.6, p<0.0001) and remembered this information one hour later (low-responsiveness group: McNemar test χ^2^ = 24.0, p<0.0001; high-responsiveness group: χ^2^ = 48.0, p<0.0001).

Despite this general pattern, the performance of both groups was significantly different. An analysis of acquisition showed that the *group*×*trial* interaction was significant (F_1,145_ = 11.3, p<0.001), thus demonstrating that the two groups behaved differently along conditioning trials. Indeed, the high-responsiveness group showed higher % of conditioned responses to the CS+ than the low-responsiveness group (Fig. 2a: F_1,145_ = 7.3; p<0.01). Responses to the CS− did not differ between groups (F_1,145_ = 0.21; NS). Differences in retention performance were also found between groups. Bees of the high-responsiveness group responded more to the CS+ than bees of the low-responsiveness group (Fisher exact test: p<0.005) while no differences were found for the CS− (NS).

To verify these conclusions, we computed for each bee and at each trial a delta value (Δ) resulting from the difference between its response to the CS+ and to the CS−. Figure 2b shows the Δ values for both groups of bees, both for acquisition and retention. Significant differences between groups were observed from the 3^rd^ trial on (3^rd^ trial: Mann-Whitney test, Z_adj_ = 2.6, p<0.01; 4^th^ trial: Z_adj_ = 3.3, p<0.001; 5^th^ trial: Z_adj_ = 2.2, p<0.05; 6^th^ trial: Z_adj_ = 2.4, p<0.02) and in retention tests (Z_adj_ = 2.8, p<0.005), thus showing that highly responsive bees learned and remembered better in the aversive discrimination task than lowly responsive bees.

### Experiment 3: Do differences in shock responsiveness underlie task specialization and different aversive learning and retention performances in guard and forager bees?

Different sucrose responsiveness are exhibited by different strains of bees specialized in different tasks within the hive [11]. Pollen foragers are highly responsive to sucrose solution including low-concentrated solutions. Nectar foragers, in contrast, are less responsive than pollen foragers, reacting only to high sucrose concentrations, thus being more selective for nectar rewards. As sucrose responsiveness is in turn correlated with learning capabilities (see above), pollen foragers learn better appetitive associations than nectar foragers [18].

Are the same trends found in the aversive modality? Do nectar foragers and guard bees differ in their shock responsiveness and do they exhibit, accordingly, different aversive learning and retention performances? To answer this question we determined shock responsiveness scores of guard and nectar forager bees of the same hive using the same procedure as above. Foragers (n = 205) were collected upon arrival at a feeder to which they were previously trained, thus ensuring that they were indeed foraging for sucrose solution. Guards (n = 151) were collected at the hive entrance after eliciting attack by means of a mechanical disturbance. After determining shock responsiveness scores of these two groups, we trained them on the next day in a differential conditioning procedure following the procedure explained above. Retention tests were again performed 1 h after the last conditioning trial.

**Figure 3:**
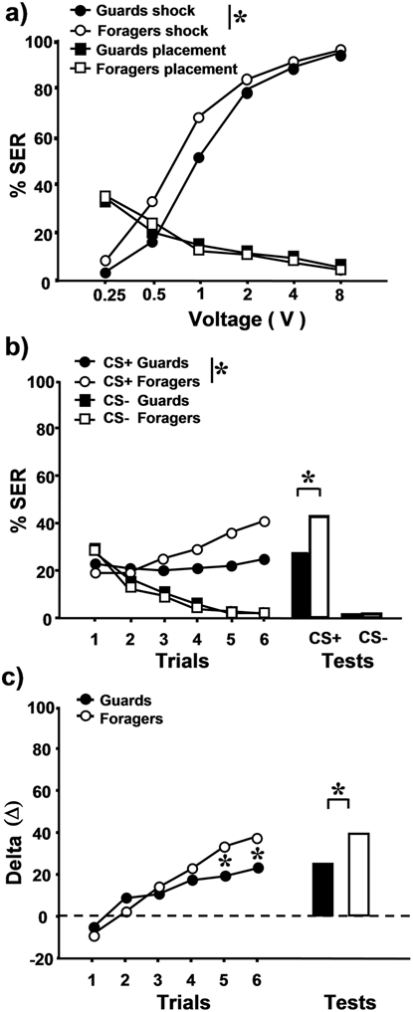
Shock responsiveness and learning and retention performances of guard and nectar forager bees. a) Guard bees (black circles; n = 151) were less responsive to a series of shocks of increasing voltage than forager bees (white circles; n = 205). Black and white squares represent the SER responses to the placements in the same setup without shock (control) of guard and forager bees respectively. b) % of SER responses of guard (black symbols; n = 105) and nectar forager bees (white symbols; n = 102) during differential SER conditioning. Circles: SER to CS+; Squares: SER to CS−. Both groups learned the discrimination between punished and non-punished odors but nectar forager bees responded more to and remembered better the CS+ one hour after conditioning (white vs. black bars) than guard bees. c) Delta value (Δ) resulting from the difference between the response to the CS+ and to the CS− along conditioning of nectar foragers (white circles) and guard bees (black circles). Foragers learned better to differentiate between CS+ and CS− and remembered better the difference (white vs. black bars). *: p<0.05.

Shock responsiveness scores of guards and nectar foragers were significantly different (Fig. 3a: F_1,354_ = 11.08, p<0.001), the responses of foragers to shocks being generally higher than those of guards, especially for lower voltages. No differences were found in responses to placements (control) trials (F_1,354_ = 0.07, NS). Thus, guards are less sensitive to electric shocks than nectar foragers.

On the second day, both groups were subjected to an olfactory differential conditioning task, as in the previous experiment. Figure 3b shows that both guards (n = 105) and nectar foragers (n = 102) learned to discriminate between the CS+ and the CS− (guards: F_1,208_ = 9.3, p<0.005; foragers: F_1,202_ = 17.0, p<0.0001) and remembered the aversive association one hour later (guards: Mc Nemar test, χ^2^ = 25.0, p<0.0001; foragers: χ^2^ = 37.2, p<0.0001). Differences in sample size between the first and second day of experiment were due to mortality and to the exclusion of bees that did not respond to any voltage and of bees that did not exhibit the unconditioned response (SER) during conditioning (see Materials and Methods). A global analysis of acquisition showed that the interaction between groups, odorants and trials was significant (F_5,1025_ = 3.8, p<0.01), thus showing that guards and nectar foragers learned the odors differently along trials. Although both groups responded similarly to the CS− during conditioning (group effect: F_1,205_ = 0.13, NS; *group*×*trial* interaction: F_5,1025_ = 0.36, NS), the evolution of responses to the CS+ were different as shown by a significant *group*×*trial* interaction (F_5,1025_ = 5.4, p<0.0001; group effect: F_1,205_ = 1.3, NS). At the end of training, nectar foragers responded significantly more to the CS+ than guards (trial 5, Fisher's exact test, p<0.05; trial 6 p<0.02). We found the same pattern of differences in the retention tests as nectar foragers remembered significantly better the CS+ than guards (Fisher's exact test, p<0.05) but did not differ in their response to the CS− (NS). As in the previous experiment, for each bee and trial we computed a delta value (Δ) as the difference between the bee's response to the CS+ and to the CS−. Figure 3c represents these delta values and confirms that nectar foragers learned better than guards to differentiate between odorants in the conditioning task from trial 5 on (trial 5: Z_adj_ =  2.3, p<0.02, trial 6 : Z_adj_ = 2.4, p<0.02). This difference was maintained in the retention tests where foragers performed better than guards (Z_adj_ = 2.2, p<0.05). We conclude, therefore, that nectar foragers and guards significantly differ in their responsiveness to electric shocks and that the more responsive, and presumably more sensitive, foragers are the ones learning and remembering better aversive associations. Although this result may appear surprising, it may be adaptive for guards to be less sensitive, and presumably more tolerant, to noxious stimuli (see discussion). Accordingly, they would assign low values to an aversive reinforcement, thus determining lower acquisition and retention performances.

## Discussion

The present work shows that the argument positing that sensitivities to different sensory stimuli are necessarily correlated [10] is not tenable. We have shown that responsiveness to sucrose does not account for responsiveness to electric shock, a stimulus with a hedonic value drastically different from that of sucrose. Thus, deducing stimulus sensitivity exclusively from sucrose sensitivity is, in any case, incautious as bees responding maximally to sugar are generally not those responding to shock. We have also shown that the notion that sensitivity to a given reinforcement translates into better learning and retention performances with such reinforcement is valid, independently of the hedonic value of the considered reinforcement. Indeed, in the same way that bees that are more responsive to sucrose learn and memorize better in olfactory conditioning protocols using sucrose as reinforcement, bees that are more responsive to electric shock also learn and memorize better in olfactory aversive conditioning, which uses electric shock as reinforcement. We show that foragers are better learners than guards in aversive conditioning, a fact that can be explained on the basis of differences in responsiveness to electric shock. Indeed, foragers are more responsive to shocks than guards as they extend their sting to a broader range of voltages.

### De-correlation between sucrose and shock responsiveness

The fact that sucrose responsiveness does not account for responsiveness to electric shock is in contradiction with previous suggestions [10] arguing that “bees who are sensitive to sucrose are also sensitive to stimuli of other modalities”. In fact, correlated responsiveness has been observed in the case of stimuli that are related to the appetitive search for food in which bees engage as foragers [21], [24], [23]. From this perspective, it seems coherent that responsiveness to odors (which are characteristic of food sources) and to light (which elicits foraging flight), as well as motor activity, are correlated in the same bees [21], [24], [23]. This variety of related sensitivities for different stimuli defines a behavioral syndrome, in this case, a “foraging behavior syndrome” [31], which can be understood as a suite of correlated behaviors reflecting between-individual consistency in behavior across multiple foraging situations [32]. Such a syndrome could also include high sensitivity to stimuli or situations so far untested like colors (better detection and discrimination performances expected in foragers highly responsive to sucrose), achromatic patterns (same as colors) and spatial memory (better spatial performances in foragers highly responsive to sucrose).

We suggest that several behavioral syndromes coexist in an insect society. Responsiveness to electric shock represents a situation that, even if it is artificial as bees do not have to respond to electric shocks in nature, allows measuring sensitivity to a noxious stimulus. From this perspective, the framework considered here is certainly distinct from that corresponding to foraging activities and thus to a foraging syndrome. A “defensive behavior syndrome” could be postulated, in which a correlated suite of defensive traits could be linked to sensitivity to electric shock. For instance, responsiveness to shock could be correlated to defensive responsiveness to Isopentyl Acetate (IPA), the main component of the sting alarm pheromone [33], 2-Heptanone, an alarming substance released by mandibular glands [34], and to intruders, represented by a moving object at the hive entrance [35]. We propose that, foraging and defensive syndromes would relate to independent, insulated modules coexisting within the same individual, and defining its tendency to act as a forager or as a defender (see below).

### Neural bases of foraging and defensive modularity

Syndrome modularity can be explained by the neurobiology of reinforcement processing in insects. Appetitive reinforcement, particularly sugar or water, is mediated by octopaminergic neurons in the insect brain. For instance, octopamine injections in the bee brain substitute for sucrose reward and induce olfactory learning [36]. Similarly, disrupting octopamine receptor function impairs olfactory learning in bees [37], probably because of the impossibility of sensing sucrose reward at a central level. In contrast, dopamine is necessary for aversive olfactory learning in insects (Drosophila: [38] Drosophila larvae: [39]; crickets: [40], [41]; bees: [30]). For instance, bees subjected to pharmacological blocking of their dopaminergic system are unable to learn the discrimination between an odorant reinforced with shock from an unreinforced odorant [30]. The involvement of octopamine in appetitive learning and memory and that of dopamine in aversive learning and memory are a widespread phenomenon occurring across insect species and sensory modalities [38]–[41]. We therefore suggest that foraging and defensive syndromes rely on separate neural systems dedicated to the processing of appetitive and aversive reinforcements, respectively. Insulation between these neural systems has been recently shown in the honeybee as bees can master simultaneously aversive (SER) and appetitive (PER) olfactory discriminations during the same conditioning session [30]. This result shows the relative independence of appetitive and aversive memories in honeybees.

### Reinforcement sensitivity and learning and memory performances

We found that the more responsive a bee is to shocks, the better it learns to associate an odorant to this noxious stimulus. This finding is consistent with the notion that reinforcement sensitivity determines learning and retention performances. In honeybees, this notion has been repeatedly demonstrated in appetitive learning using sucrose as reinforcement [17]–[22]. The more responsive a bee is to sucrose, the better it learns and memorizes in appetitive olfactory and tactile learning protocols. These findings correspond to the role given to reinforcement salience or strength in learning theories. For instance, the Rescorla and Wagner model, developed for classical conditioning [42], assumes that learning directly depends on the salience and intensity of both the conditioned and the unconditioned stimulus (the reinforcement). Clearly, the more salient a reinforcement is, the better the learning performance. Salience depends on physical properties of the stimulus but also on internal, subjective evaluation. In our case, such evaluation is reflected by the animal's responsiveness to the tested reinforcement. Scheiner et al. [22] have shown that it is possible to suppress differences in appetitive learning between bees having different sucrose responsiveness if they are provided with an “equal subjective reward” (i.e. a reward eliciting the same level of responses in two bees having different sucrose responsiveness). The same should occur in aversive learning. Training bees with different shock responsiveness with different voltages chosen to elicit the same levels of SER should result in similar aversive learning and retention performances.

### Shock responsiveness and aversive learning in guards and foragers

Like sucrose responsiveness, responsiveness to a particular noxious stimulus could also be linked to a defensive division of labor. We therefore compared shock responsiveness in guards and nectar foragers of the same hive. We found that guards are less responsive to shocks than nectar foragers and that they learn less efficiently in SER conditioning. This result seems to be contradictory with the fact that guard bees protect the hive from intruders and robbers and are exposed to multiple aversive experiences. It is, however, consistent with the finding that nectar foragers are less responsive to sucrose than pollen or water foragers and that their learning performances are lower during an appetitive conditioning [14]. In fact, both scenarios could be reconciled by considering that both nectar foragers and guards are particularly selective for the stimulus intensities to which they should respond in nature. In other words, nectar foragers respond only to the highest sucrose concentrations (and are therefore globally less responsive if one considers the spectrum of concentrations tested) and, similarly, guards respond only to the highest voltages assayed, thus being more tolerant to lower noxious stimulus levels. Such low sensitivity of guards to noxious stimuli may indeed be adaptive for honeybees, as defensive responses are costly for the colony (especially when recruitment takes place), and such a defensive response should not be triggered by aggressions of low intensity, but rather by rather dangerous situations for the colony. Another proof for this stimulus selectivity is the fact that although Africanized honeybees are known to be more aggressive than European honeybees [43], the former are less responsive, i.e. more tolerant, to a noxious stimulus (electric shocks) than the latter [44].

Again, neural-based explanations could account for the difference found between guards and foragers in shock responsiveness and aversive learning and retention. Dopamine levels in the bee brain depend on age [45], [46] so that older bees have more dopamine in their brains. Foragers, which are generally older than guards, would be therefore more prone to learn about aversive associations than guards, as shown by our work. Dopamine levels also depend on contact with queen mandibular pheromone (QMP), a substance produced by the queen, which has priming and acute effects on social control within a bee colony [47]. More precisely, younger bees, which come closer to the queen and to QMP, present lower levels of dopamine in their brains while older ones, which tend to move outside the hive and thus to become more distant from QMP, present higher levels of dopamine [48]. It can therefore be predicted that foragers should exhibit better aversive learning performances than guards, which is exactly what we found here. In this scenario, impaired aversive learning would be due to the incapacity to signal aversive reinforcement appropriately due to low dopamine levels.

Coincident with this explanation, Uribe-Rubio et al. [49] showed that Africanized guard bees are faster to sting in response to an electric shock than younger, nest bees. Also, Paxton et al. [50] found that older European bees sting at a lower voltage than young bees. A single-cohort experiment [51] in which bees of the same age could be biased to perform different tasks could allow deciding whether dopamine levels, and thus responsiveness to electric shock, are the consequence of task specialization and of queen proximity, or result from age, independently of the task performed. In our case, we propose that guards are less responsive to shocks and learn less efficiently in SER conditioning than foragers, because they are younger, come eventually closer to the queen, and present therefore lower dopamine levels in their brains. To check this hypothesis, dopamine levels of guards and nectar foragers could be compared using HPLC. Furthermore, SER varies between patrilines of the same hive [29], thus indicating a genetic contribution of this behavior to intra-colonial variation. It could be therefore interesting to test the effect of patrilines in our experiments, in particular whether guards belong to a particular patriline while foragers to a different one.

### Reconsidering threshold theory and division of labor

One of the main conclusions of our work is that bees that exhibit higher responsiveness for a noxious stimulus do not necessarily specialize in a defensive task such as guarding. As mentioned above, guards responded only to the highest voltage levels compared to foragers. This result has been repeatedly verified in the appetitive modality: bees that are highly responsive to sucrose solution are not nectar but pollen foragers, while bees responding only to highest sucrose concentrations are nectar foragers. It seems therefore necessary to reconsider how the threshold theory of division of labor is formulated. It is commonly said that animals with *lower* thresholds for a given stimulus will tend to specialize in tasks involving such stimulus. The examples of nectar foragers [21], and now of guards (our work), show that if thresholds are measured in terms of responsiveness to a range of various intensities, this formulation is inappropriate. Indeed, nectar foragers are those bees exhibiting not the lowest but the *highest* response thresholds to sucrose. Similarly, guard bees are those with the *highest* response thresholds to electric shock. It seems therefore that what is relevant to consider when measuring response thresholds is the selectivity of an animal towards intensity variations of a given stimulus.

All in all, the threshold theory provides an appropriate framework to understand division of labor but requires accurate formulations. Higher response thresholds are not necessarily contradictory with task specialization as they may reflect higher selectivity for a given stimulus, a factor necessary for specialization to occur and develop, and that may be adaptive in particular situations.

## Materials and Methods

*Apis mellifera* bees collected from a hive were brought to the laboratory and chilled on ice for 5 min until they stopped moving. They were then harnessed on individual holders designed for aversive conditioning [30]. After 1-hour rest, bees were exposed to a succession of 6 electric shocks of increasing voltage (0.25, 0.5, 1, 2, 4 and 8 V) corresponding to a logarithmic series [25]. We assessed the bees' SER and thus the thresholds of responsiveness to this aversive stimulus. In order to avoid sensitization of SER along our stimulation sequence, we interspersed placement trials between each voltage trial, in which bees were placed in the stimulation setup without receiving any shock. During such placement trials, we recorded whether bees exhibited SER during the 2 s corresponding to the timing of the electric shock in voltage trials. Consecutive tests were separated by 2 min. Full extension of the sting was scored as 1. No response or partial ones were scored as 0 [30]. The aversive responsiveness score of each bee was calculated as the sum of all responses made along the whole scale of voltages tested. For example, a bee extending its sting from 0.5 to 8 V has an aversive score of 5 as it responds to five consecutive voltages. Bees starting to respond to a given voltage and not responding to higher subsequent ones were not included in the analyses as their aversive score would be meaningless. Such bees were however very few and represented only 4.04% of all bees tested in this study (n = 1016).

### Experiment 1: Do sucrose and shock responsiveness correlate?

In one group of bees we measured shock responsiveness in a fjrst phase and sucrose responsiveness in a second phase; in another group, we did the opposite in order to exclude possible sequential effects. A 1-h rest period was inserted between the two experimental phases. Shock responsiveness was measured as explained above. Sucrose responsiveness to sucrose solution of increasing concentration was measured along 6 successive antennal stimulations (0.1, 0.3, 1, 3, 10 and 30%; weight / weight) corresponding to a logarithmic series [11]. We assessed the bees' PER and thus the thresholds of responsiveness to this appetitive stimulus. In order to avoid sensitization of PER along our stimulation sequence, we interspersed water stimulation of the antennae (water trials) between each sucrose trial [11]. Consecutive tests were separated by 2 min. Full extension of the proboscis was scored as 1. No response or partial ones were scored as 0. The appetitive responsiveness score of each bee was calculated as the sum of all responses made along the whole scale of sucrose concentrations tested. For example, a bee extending its proboscis from 0.3 to 30% has an aversive score of 5 as it responds to five consecutive concentrations. Again, bees with inconsistent responses were not considered for analyses (11.01%, n = 227). At the end of the experiment, sucrose and shock responsiveness scores were available for each individual, therefore allowing correlative analyses between these variables.

### Experiment 2: Does shock responsiveness determine aversive learning and retention performances?

On the first day bees' shock responsiveness scores were determined as above. Bees not exhibiting any SER to the succession of voltages tested (0 score) were excluded from the experiment as it would be impossible to condition them in the absence of unconditioned responses. Bees were divided in two groups according to their scores, a low-responsiveness group (scores 1 to 3) and a high-responsiveness group (scores 4 to 6). They were identified by means of a color spot on the abdomen. A different color was assigned to each group. After marking them, bees were freed and placed in a box containing food and water at will and maintained at 25°C until the next day. On the second day, bees were harnessed again and after 2 h rest they were subjected to olfactory conditioning of SER [30].

Bees were trained in a differential conditioning procedure (one reinforced odorant or CS+ vs. a non-reinforced odorant or CS−) using 1-hexanol and 1-nonanol (Sigma Aldrich, Deisenhofen, Germany). Five µl of pure odorant were applied onto 1 cm^2^ filter paper pieces placed into a 20 ml syringe, thus allowing odorant delivery to the antennae. Each odorant was delivered for 5 s. An air extractor placed behind the bee prevented odorant accumulation, as well as possible contamination by pheromone release. The voltage used was 7.5 V, delivered during 2 s, which is the optimum for aversive conditioning (unpublished data from our group).

Half of the bees received a shock on 1-hexanol trials and no shock on 1-nonanol trials while the reversed contingency was used for the other half. Both groups were conditioned along 12 trials (6 reinforced and 6 non-reinforced) in which odorants were presented in a pseudo-random sequence (e.g. ABBABAABABBA) starting with odorant A or B in a balanced way. Each conditioning trial lasted 1 min. The bee was placed in the stimulation site in front of the air extractor and left for 20 sec before being exposed to the odorant paired with the electric shock. The electric shock started 3 sec after odorant onset and finished with the odorant. The bee was then left in the setup for 35 sec and then removed. The intertrial interval (ITI) was always 10 min. Retention tests were performed 1 h after the last conditioning trial and consisted of presenting in a random order the CS+ and the CS− without reinforcement. We quantified SER during the presentation of the odorants (conditioned responses) and during the shock (unconditioned responses]. Bees not responding to the shock were not used for the analyses (3.9%, n = 153).

### Experiment 3: Do differences in shock responsiveness underlie task specialization and different aversive learning and retention performances in guard and forager bees?

We selectively collected foragers and guard bees from the same hive. Nectar foragers were collected at a feeder containing 30% sucrose solution, to which they were previously trained. The feeder was placed 100 m away from the hive. Bees were collected in glass vials upon arrival at the feeder and before they started feeding. Guards were collected at the hive entrance after eliciting attack by means of a mechanical disturbance produced by a stick located at the hive entrance. Only the very first bees exiting and attacking during the first minute following disturbance were collected by means of a transparent Plexiglas box.

Guards and foragers were individually harnessed and subjected to the procedure of Experiment 2 (i.e. assessment of their shock responsiveness score on the first day and conditioning and retention tests on the second day).

### Statistical analysis

In Experiment 1, correlation between shock and sucrose responsiveness in the same bees was assessed using Spearman correlation analysis. In order to test whether the phase of testing (first or second) affected appetitive or aversive responsiveness, performances were compared between phases using repeated-measurement ANOVA. Studies based on Monte Carlo simulations have shown that it is permissible to use ANOVA on dichotomous data such as those from PER or SER only under controlled conditions, which are met by our experiments [52]. In Experiments 2 and 3, repeated-measurement ANOVA was used to analyze acquisition during trials both for between- and within-group comparisons. Performances in the retention tests were analyzed by means of a Mc Nemar test for within-group comparisons and with Fisher's exact test for between-group comparisons. We quantified differentiation during acquisition by computing for each bee and each trial a delta value resulting from the difference between its CS+ and its CS− responses. Thus, delta could take values of −1, 0 or 1. Mann-Whitney tests were used to evaluate differences of deltas between groups.
